# The effect of a video tutorial to improve patients’ keratoconus knowledge – a randomized controlled trial and meta-analysis of published reports

**DOI:** 10.3389/fopht.2022.997257

**Published:** 2022-10-24

**Authors:** Chiara Sommer, Lucas M. Bachmann, Armin Handzic, Katja C. Iselin, Frantisek Sanak, Oliver Pfaeffli, Claude Kaufmann, Michael A. Thiel, Philipp B. Baenninger

**Affiliations:** ^1^ Medical Faculty, University of Zurich, Zurich, Switzerland; ^2^ Medignition Inc. Research Consultants Zurich, Zurich, Switzerland; ^3^ Department of Ophthalmology, Cantonal Hospital of Lucerne, Lucerne, Switzerland

**Keywords:** improve knowledge, keratoconus, educational intervention, video information, video tutorial, randomized controlled trial

## Abstract

**Objective:**

To investigate whether a video tutorial, highlighting important aspects of keratoconus provided prior to a scheduled follow-up consultation, has a specific effect on patients’ knowledge after the consultation.

**Methods and Analysis:**

Single center, randomized controlled trial registered on ISRCTN registry (number ISCTN75317089, https://doi.org/10.1186/ISRCTN75317089). Consenting eligible keratoconus patients were randomly assigned to either receive a conventional face-to-face consultation (control group) or to an additional video tutorial (interventional group) on definition, risk factors and treatment options provided prior to the consultation. The main outcome measure was the difference of knowledge assessed by a questionnaire after the consultation. Of each participant, clinical characteristics, highest educational level and medical background were obtained. We also performed a meta-analysis of published reports assessing knowledge improvement by video-based patient education.

**Results:**

We assigned 22 patients to the interventional and 21 patients to the control group. Mean age was 29.0 years (SD 11.6), 8/43 (18.6%) were female and median disease duration was 2.5 years (interquartile range: 2-5years). Compared to the control group, knowledge was 12.0% (95%CI: 5.8%-18.2%; p<0.001) higher in the interventional group. Subjects with a university degree scored 6.8% (95%CI: 3.8%-13.3%; p=0.038) higher. There was no interaction between video information and university degree. Other parameters were not associated with patient knowledge. The meta-analysis of 566 subjects enrolled in 6 studies revealed a standardized mean difference in favor of video-based education of 0.47 (95% CI: 0.30-0.64; p<0.004)

**Conclusion:**

The results suggest that supplementary video information embedded in the clinical management of keratoconus, helps conveying relevant disease knowledge.

## Introduction

Keratoconus is a progressive, ectatic corneal disease with abnormal corneal thickness distribution and clinical noninflammatory corneal thinning (1). Treatment focuses on stopping the progression with collagen cross-linking and visual rehabilitation with glasses, hard contact lenses or corneal transplantation (1). The last two decades have revolutionized the knowledge about the diagnosis and treatment of the disease. However, it is not only important to evaluate new diagnostic technologies and improve treatment options for patients, but also to focus on patient-oriented care and facilitate joint decision-making.

Shared decision-making can guide patients who face difficult treatment decisions where risks and benefits need to be weighed against each other, taking into account patient goals and preferences (2). Digital solutions play an increasing role for the delivery of multifaceted, multi-media education and empower patients to develop their decision making skills (3), however little is known about their effect on patients’ knowledge. Knowledge deficiency promotes patient’s concerns and fears, and leads to unrealistic expectations about the course of the disease (4). A recent study showed a substantial mismatch between caregivers’ expectations of keratoconus patients’ knowledge regarding their condition (5). We are unaware of studies assessing the effects of training or education on the improvement of knowledge in patients with keratoconus. Therefore, this study investigated the benefit of a video tutorial prior to an ophthalmologic consultation regarding patients’ keratoconus knowledge within a randomized controlled trial setting. Furthermore, we performed a meta-analysis of published reports assessing knowledge improvement by video-based patient education.

## Materials and methods

### Ethics

The local Ethics committee of Lucerne reviewed the protocol of this study and waived the need for IRB approval (Project ID: 2020-00164). The IRB confirmed that this study was in line with the general ethical principles for research involving human beings (cf. Art. 51 para. 2 Human Research Act). The study was conducted according to the standards of good clinical practice and the ethical principles for medical research involving human subjects as outlined in the Declaration of Helsinki (6).

### Study design

This study is a single-center, single blinded, randomized controlled trial and was conducted at the Cantonal Hospital of Lucerne, Switzerland. The intervention group received a video tutorial prior to a consultation with a for the study allocation blinded physician regarding the knowledge of keratoconus. We compare the effects in the intervention group with the effects of a control group of the same size, which receives the usual information during the consultation. This trial has been registered at ISRCTN registry (ISCTN75317089) by January 13, 2020. The reporting of this randomized trial adheres to the tenets of the CONSORT statement (7).

### Study population and recruitment

Potential eligible patients presenting for keratoconus consultation at the corneal clinic were informed about the existence of the study. The only inclusion criteria were previous diagnosis of keratoconus on at least one eye and the ability to provide an informed consent. The diagnosis of keratoconus was confirmed topographically using Pentacam. Exclusion criteria were inability to follow the procedure of the study due to i.e. language problems, psychological disorders or dementia, patients aged under 18 years, patients under tutelage, previous enrollment into the current study and enrollment of the investigator, his family members, employees or other dependent persons. We enrolled patients willing to participate in the study in a prospective and consecutive manner. Written informed consent was obtained from all participants. Three board-certified senior staff conducted the consultations.

### Investigations

Participating patients were enrolled by the clinical trial study nurse and randomized either to the interventional group or controls. Patients of the intervention group received a five minutes video tutorial covering the most important aspects of keratoconus. The control group attended the visit without additional information prior to the consultation.

### Randomization and blinding of caregivers

We pre-stratified for duration since keratoconus diagnosis (< 5 years versus ≥ 5 years) and contact lens wear (yes versus no), since the disease knowledge might increase with disease duration and contact lens wear. Randomization was made with a 1:1 allocation ratio and using blocks of 2 and 4. The randomization list was kept at the trial center (concealed) and caregivers at the consultation were blinded to the allocation.

### Intervention

The video tutorial shows different animated scenes giving the same standardized information on keratoconus as the treating ophthalmologist gives in regular face-to-face consultation. The material was developed in collaboration with members of the medical faculty of the University of Zurich and a group of clinical experts at the eye clinic of Cantonal Hospital of Lucerne in January 2020. The duration of the video tutorial takes about five minutes and can be accessed at: www.youtube.com/watch?v=9oWeP137xnI [Video in German language]. A translated form of the instruction text in the video tutorial is available at **Additional File 1**.

### Outcome assessment

The questionnaire was developed using questions from a different existing questionnaire (5) and also contained questions specific to this study. The final questionnaire contained six multiple choice questions with five statements each that could either be correct or false. The maximum score is 30 and the minimum score is 0.

After the consultation, all patients received the paper-based questionnaire in German language (available in translated form in the **Additional File 2**), assessing knowledge about definition, risk factors, triggers, symptoms and treatment options.

From each participant, information on age, gender, highest educational degree and medical background were obtained. We also asked for the duration since diagnosis and their current treatment of keratoconus. Ophthalmic findings, including uncorrected and best corrected visual acuity, manifest refraction, maximal keratometry (Kmax), thinnest corneal pachymetry and Belin-Ambrosio D-value (from Pentacam) and the classification of keratoconus per eye using the Amsler-Krumeich classification were also extracted of each participant.

### Study outcome measures

The primary study objective was to investigate the percentage difference of knowledge between patients of the intervention and the control groups as assessed with the questionnaire. Secondary objectives were to assess effect modification by age, gender, highest educational degree, medical background and duration of keratoconus and to investigate the effect of ophthalmic findings such as visual acuity, steepness of the cornea (Kmax), the extent of ectasia (using the Belin-Ambrosio Deviation score) and type of previous surgical treatments.

### Determination of sample size

This study was made under the assumption that the average proportion of correct replies in the intervention group is 27/30 (90%) and 15/30 (50%) in the control group. We further assumed that the interventional group would always have a better performance than the control group. We therefore accepted a one-sided alpha error of 5 percent. Finally, the beta error was set at 20 percent (power of 80 percent). Following these assumptions, the number of participants per group is 20, i.e. the total number of patients participating in this study is 40.

### Statistical analysis

The analysis was made on the intention-to-treat principle. Summaries of continuous variables are given with means and standard deviations (SD) or medians and interquartile ranges (IQR). Dichotomous variables are summarized with percentages. We counted the cumulative number of correct replies and divided this number by 30 to obtain the percentage of correct replies per subject. Indicator variates for duration since keratoconus diagnosis (< 5 years versus ≥ 5 years) and contact lens wear (yes versus no) were made.

For the primary outcome, we quantified the association between the group allocation and the percentage of correct replies using a univariate model. Using multivariate linear models, we examined the influence of age (interval scaled), gender (female; male) and highest educational degree (university; else), severity of keratoconus (Amsler-Krumeich classification), medical background and history of surgical treatment knowledge within the randomized groups. Product terms were used to assess interaction between random allocation and duration since keratoconus diagnosis, university degree and contact lens wear. A p-value of less than 5 percent was considered statistically significant.

### Meta-analysis

An electronic search revealed a recently published systematic review on video-based media as patient education tool in ophthalmology (8). Additionally, a further study assessing video-presented information about excimer laser treatment was included (9). We assessed only studies with available primary data on knowledge improvement allowing to calculate performance characteristics (10–13). As the individual studies used different instruments, we performed the meta-analysis using standardized mean differences. By using this statistic, we assumed that the differences in standard deviations among studies reflected differences in measurement scales and not real differences in variability among study populations

We performed the meta-analysis excluding and including the current report to assess the impact of the present study on the pooled estimate. We performed the meta-analysis using a fixed and a random effects model and provide the results of the fixed effects model providing a conservative pooled estimate. Analyses were performed using the Stata metan routine. We performed the analysis using the Stata 16.1 statistical software package (StataCorp, 4905 Lakeway Drive, College Station, Texas 77845 USA).

## Results

### Patients’ characteristics

**Figure 1:**
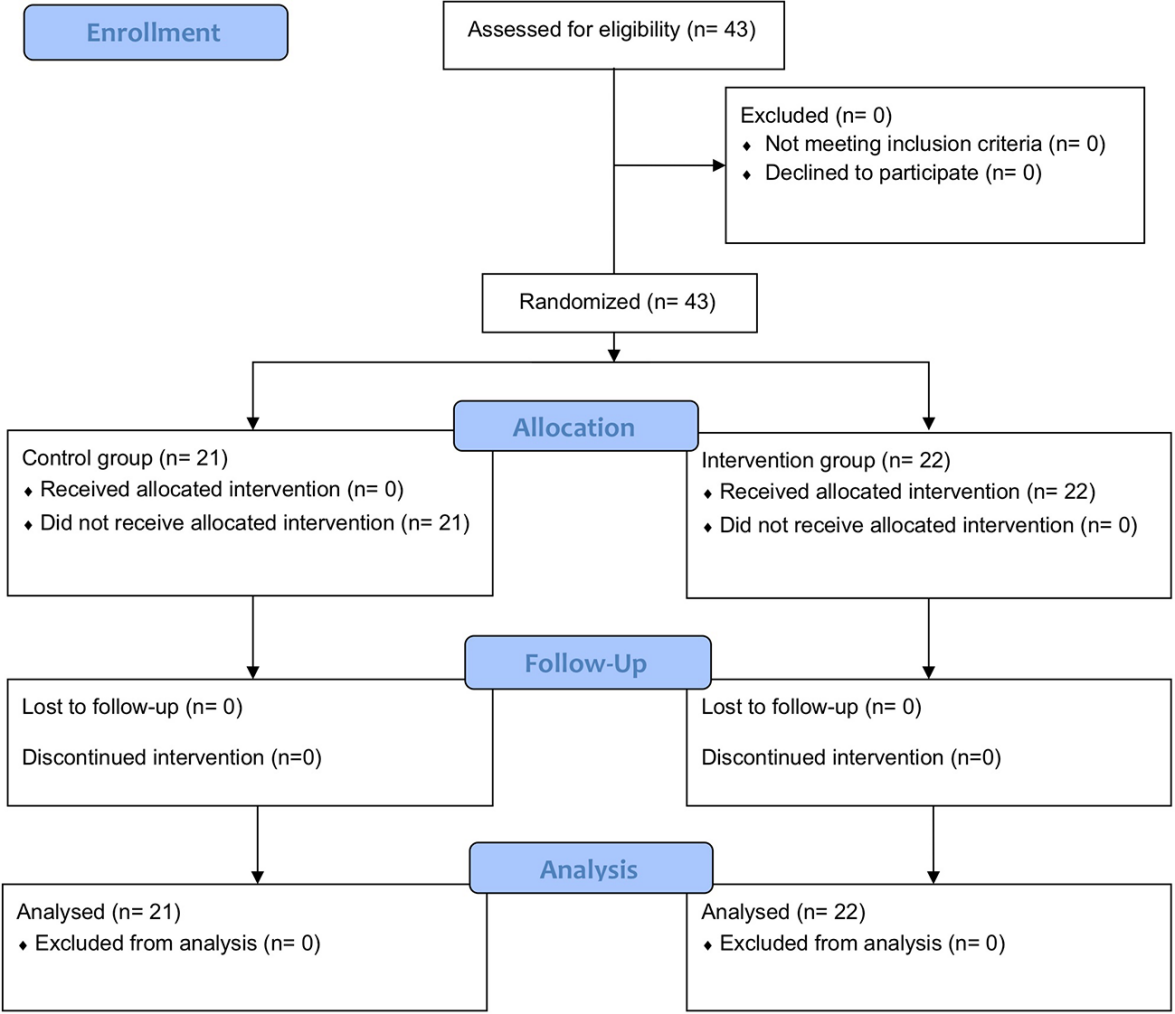
CONSORT Flow Diagram.

During a recruiting period between May and July 2020, we consecutively enrolled all consenting patients into either the interventional group (n=22) or the control group (n=21). Participant flow is described on the Consort Flow Diagram (**Figure 1**). The patient allocation to each of the three board-certified senior staff was balanced and each eyecare professional assessed a similar number of patients. Both groups were comparable at baseline and all variables were normally distributed. Baseline characteristics are listed in **Table 1**.

**Table 1:** Patients’ characteristics and score values.

Patients’ characteristics		interventional group (n=22)		control group (n=21)	
**female**		18%		19%	
**university degree**		30%		43%	
**medical background**		14%		10%	
**previous surgery**		68%		67%	
**Contact lens use**		50%		62%	
		**mean**	**SD**	**mean**	**SD**
**age**		31.09	12.17	26.86	10.77
**UCVA (logMAR) right eye**		0.64	0.62	0.68	0.47
**UCVA (logMAR) left eye**		0.74	0.62	0.75	0.59
**BCVA (logMAR) right eye**		0.07	0.31	0.13	0.24
**BCVA (logMAR) left eye**		0.09	0.30	0.04	0.13
**manifest sphere right eye**		-0.80	2.38	-0.83	3.74
**manifest sphere left eye**		-1.07	3.18	-0.46	2.91
**manifest cylinder right eye**		-2.07	1.67	-2.58	1.91
**manifest cylinder left eye**		-2.80	2.39	-2.79	2.49
**Kmax right eye**		54.03	11.78	57.63	8.59
**Kmax left eye**		53.45	6.02	54.87	9.22
**thinnest corneal pachymetry** **right eye**		467	57.1	446	89.5
**thinnest corneal pachymetry** **left eye**		469	40.5	483	51.0
**Amsler-Krumeich classification**					
**Right eye**	1	16	72.7%	11	52.4%
	2	3	13.6%	7	33.3%
	3	1	4.5%	1	4.8%
	4	2	9.1%	2	9.5%
**Left eye**	1	15	68.2%	12	57.1%
	2	3	13.6%	8	38.1%
	3	4	18.2%	0	0.0%
	4	0	0.0%	1	4.8%
**Bellin-Ambrosio score**					
**Right eye**		7.94	7.07	10.45	8.05
**Left eye**		7.63	4.17	6.75	3.66
**total score**		25.41	2.89	21.81	3.12
**score percentage** **(total score/30)**		84.7%		72.7%	

Mean overall patient age was 29.0 years (SD 11.6; range 18-51). In the interventional group, mean age was slightly higher (31.09 years (SD 12.17)), whereas mean age in the control group was slightly, albeit not significantly lower (26.86 years (SD 10.77); p = 0.235). Overall, 8 out of 43 participating patients (18.6%) were female without difference between the two groups (p= 0.624). Median disease duration was 2.5 years (interquartile range: 2-5years).

### Patients’ knowledge about keratoconus

Compared to the control group, knowledge was 12.0% (95%CI: 5.8%-18.2%; p<0.001) higher in the interventional group. We did not find any difference regarding knowledge in subjects with shorter versus longer disease duration. There was also no difference in effects seen if patients were wearing contact lenses or not in neither exposure group nor between exposure groups.

### Influence of patient demographic and ophthalmic findings on knowledge

Multivariable analysis showed that keratoconus knowledge was independent of age and gender within the randomized groups. Subjects with a university degree scored 6.8% (95%CI: 3.8%-13.3%; p=0.038) higher. Participants with a higher severity of keratoconus (0 = 0.466), medical background (p= 0.674) or history of surgical treatment (p= 0.916) had not a higher knowledge on keratoconus.

### Meta-analysis of published reports

**Figure 2:**
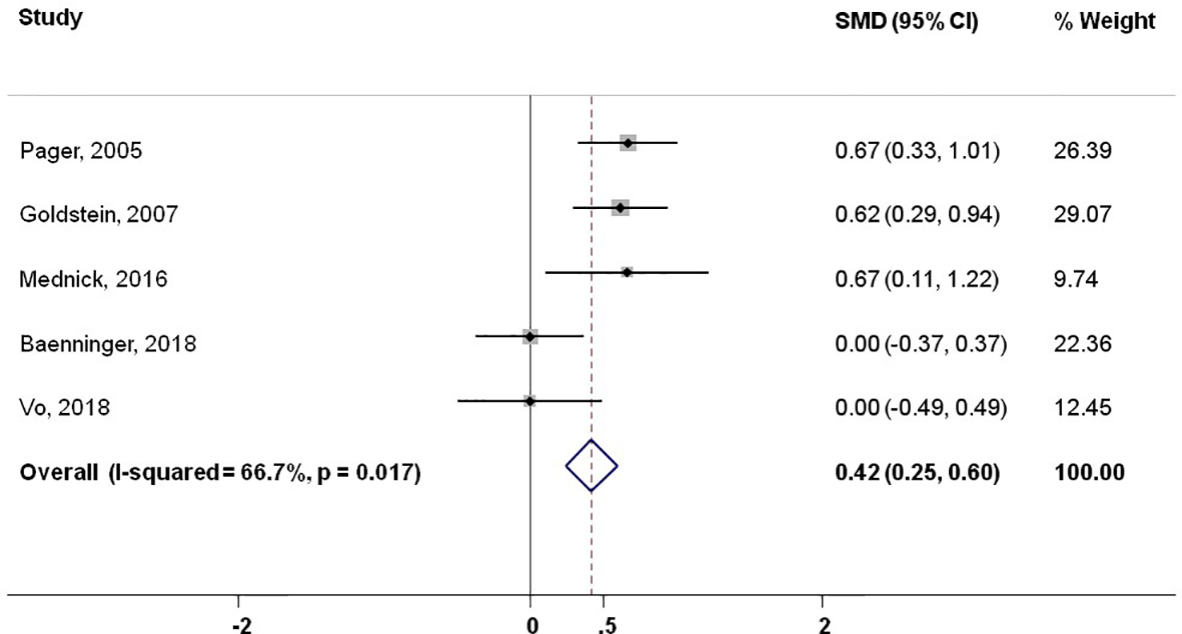
Meta-analysis of published reports showing standardized mean difference excluding present study.

**Figure 3:**
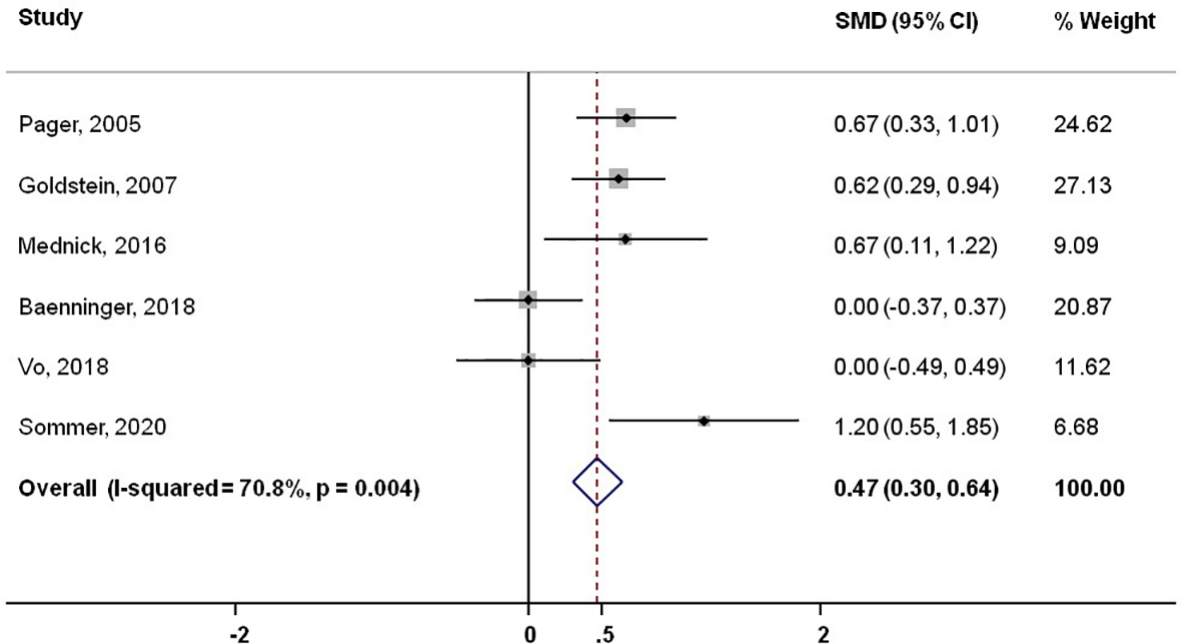
Meta-analysis of published reports showing standardized mean difference including present study.

Six studies revealed primary data on knowledge improvement by video-based media. This exploratory meta-analysis enrolled 566 patients. The study results were homogeneous regarding the direction of effect in favor of video-based patient education. The standardized mean difference in favor of video-based education of all studies was 0.47 (95% CI: 0.30-0.64; p<0.004). The pooled estimate when excluding the present study was 0.42 (95%CI: 0.25-0.60; p<0.017). The forest plots of the two analyses are shown in **Figures 2**, **3**.

## Discussion

### Main findings

This is the first randomized controlled trial evaluating the effects of a video tutorial on patients’ knowledge about keratoconus. We showed that the video tutorial significantly improved knowledge by 12% when compared to a standard consultation and was independent of age, gender, stage of keratoconus and history of surgical treatment. Participants with a university degree showed a higher knowledge by 6.8%, but medical background did not improve keratoconus knowledge.

### Results in context of the existing literature

The positive impact of patient education on patient outcomes is well established and its impact on health outcomes estimated up to 50-80% (14). Numerous digital innovations in health information technology are thereby empowering patients to assume a more active role in managing their chronic condition (15). In our study, we found a positive effect of a patient education in terms of knowledge improvement in keratoconus patients. This stands in line with ophthalmic studies assessing knowledge improvement in patients being counseled for cataract surgery (16) or influence on knowledge improvement on choice of treatment in optic neuritis management (17). Furthermore, several publications in the field of glaucoma emphasized the importance of patient knowledge improvement on treatment satisfaction (18) and treatment adherence (19, 20) with a positive effect over a 1 year follow-up (21). In the field of keratoconus, general awareness campaign such as the Violet June were started due to the evidence that lack of information or misinformation about the disease cause more suffering than the disease itself (22).

Farwana et al. (8) showed in their systematic review a significant improvement of patient comprehension using video-based media in 5 of 7 studies (71%). Our meta-analysis expands on these results by clearly demonstrating a positive effect of video-based interventions to improve patient knowledge independent of the way in which knowledge is assessed. It also shows a clear concordance of our results with the existing literature.

A recent randomized trial invested the impact of type and style of information material on glaucoma knowledge (23). Material written in simple language and containing illustrations was clearly superior to material requiring higher reading level without illustrations. Our study expanded on these findings and designed the intervention using animated illustrations and a minimum of text in a video format. Interestingly, the beneficial effects of simpler formats were similar in both studies indicating that layout and language effects reach a maximum beyond which improvements are difficult to achieve.

A review on health economic evaluations of patient education interventions for people living with chronic illness, such as keratoconus, demonstrated that patient education interventions are not only effective to improve patient knowledge but also to cut costs due to fewer hospital visits, benefits in terms of quality-adjusted life years and reduced loss of production (24).

### Strengths and limitations

To the best of our knowledge, this is the first randomized controlled trial investigating a video tutorial to improve keratoconus knowledge. The design was single blinded excluding the possibility that patients in the control group received more attention during the consultation than in typical clinical setting. Second, randomization worked well and there were no relevant differences for prognostically relevant parameters across the treatment groups. Finally, all patients completed the study and we had no drop-outs.

What are the limitations of this study? We used a non-validated questionnaire assessing patient’s knowledge of keratoconus. As there was no standard and valid questionnaire available, we designed one according to published recommendations (25). The questionnaire only fulfilled the element of face validity (26), which is an important but not a sufficient element of questionnaire development. However, the questionnaire was sufficient to assess patient knowledge in the control and interventional group. Moreover, it can be argued that health literacy is important (27) and we cannot rule out that the level of understandability was appropriate to all patients (28). However, in view that a complete understanding of the benefits and risks of the intervention is pivotal, the knowledge levels found in this study are reassuring.

Finally, it could be argued that the study was too small to draw firm quantitative conclusions. While positive effects of the video tutorial were statistically significant, we do not know, whether the knowledge difference of 16 percent points is also clinically meaningful. Furthermore, the power of this study (i.e. the ability to detect effect differences between groups when they are in fact present) was only 9% due to the difference found between groups. Moreover, considering the limited power of this study, statistical interpretation of results within subgroups is very limited. Given this difference, the required number of participants for each group would be 203 when targeting at a power of 80 percent. Therefore, to contextualize our findings in the light of previous research, we also conducted a meta-analysis with over 500 participants. Reassuringly, the results of this analysis were in line with those of our, underpowered study. Nevertheless, it can be argued that due to the brief reporting, interpretation of meta-analysis results is limited.

### Implications for practice and further research

Our study results may be extrapolated to other conditions in ophthalmology or other medical disciplines. We believe that the use of additional educational interventions such as video tutorials have the potential to increase patient knowledge and therefore lead to a better understanding of the condition, which supports shared decision-making. The prior sending of educational material allows to strengthen the patient-doctor relationship and empowers the patient to have a conversation at eye level. However, these results need to be further validated for i.e. an elderly population, as most patients in this study have been young and may have been more susceptible to modern educational measures in comparison to elderly patients (29). Furthermore, our study setting did not allow to discriminate between recall and knowledge as we asked the questionnaire immediately after the consultation. We therefore propose a follow-up study twelve months after the initial assessment using an adapted questionnaire to assess knowledge difference between the two groups. Moreover, it would be important to verify if the increase in knowledge was clinically significant in the daily life of the patient and in the management of the disease.

### Conclusions

The results suggest that **Supplementary Video** information embedded in the clinical management of a chronic disease like keratoconus, helps conveying relevant disease knowledge beyond the possibilities of a conventional consultation, even in patients with a long medical history. The results of an exploratory meta-analysis revealed that the results of this study concord with previous reports including more than 500 subjects, assessing the impact of video-based education on patients’ disease knowledge. We suggest that including a video format to convey clinically relevant information, supports shared decision making as it raises patients’ disease understanding and enable discussions at eye level with their caregivers.

## Data availability statement

The datasets used and/or analyzed during the current study are available from the corresponding author on reasonable request. The datasets generated and/or analysed during the current study are not publicly available due institutional policy but are available from the corresponding author on reasonable request.

## Ethics statement

The studies involving human participants were reviewed and approved by ethic committee of Lucerne. The patients/participants provided their written informed consent to participate in this study.

## Author contributions

CS data acquisition, writing of manuscript. LB data analysis and interpretation, writing of manuscript. AH data acquisition. KI data acquisition. FS data acquisition. OP writing of manuscript. CK substantial revision. MT substantial revision. PB Conception of work, substantial revision. All authors contributed to the article and approved the submitted version.

## Conflict of interest

Author LB was employed by medignition Inc. Research Consultants Zurich.

The remaining authors declare that the research was conducted in the absence of any commercial or financial relationships that could be construed as a potential conflict of interest.

## Publisher’s note

All claims expressed in this article are solely those of the authors and do not necessarily represent those of their affiliated organizations, or those of the publisher, the editors and the reviewers. Any product that may be evaluated in this article, or claim that may be made by its manufacturer, is not guaranteed or endorsed by the publisher.

## Supplementary material

Additional file 1 Translated text of video tutorial.

Additional file 2 Study questionnaire.
