# Supplementary material for: The effect of a video tutorial to improve patients’ keratoconus knowledge – a randomized controlled trial and meta-analysis of published reports
Source: Front Ophthalmol (Lausanne). 2022 Oct 24;2:997257. doi: 10.3389/fopht.2022.997257 (PMC11182323; doi:10.3389/fopht.2022.997257)
Supplement: Additional file 1 — Translated text of video tutorial. [file DataSheet_1.docx]

**Educational intervention**

*Intervention*

The video tutorial shows different animated scenes giving the same standardized information on keratoconus. The material was developed in collaboration with members of the medical faculty of the University of Zurich and a group of clinical experts at the eye clinic of Cantonal Hospital of Lucerne in January 2020. The duration of the video tutorial takes about five minutes and can be accessed at: [www.youtube.com/watch?v=9oWeP137xnI](http://www.youtube.com/watch?v=9oWeP137xnI)www.youtube.com/watch?v=9oWeP137xnI [Video in German language].

*English translation of video tutorial text*

Dear keratoconus patients and relatives

In this video we explain important facts about keratoconus with the aim of increasing your knowledge.

Capter One: What is a keratoconus?

Keratoconus is a progressively worsening eye disease that causes the cornea to bulge and thin, resulting in irregular corneal curvature.

Usually, keratoconus occurs in adolescence. The course of the disease varies greatly from person to person. Up to the age of about 35 years, a progression of the disease and thus an increase in corneal changes and subjective complaints must be expected. After that, a stable condition often develops. The progressive change can occur in stages, e.g. a more rapid progression can be observed in childhood or during pregnancy. Generally, both eyes are affected by the disease, but to different degrees. The course of the keratoconus disease is also often not equally severe in both eyes.

Capter two: Causes, risk factors and potential triggers?

The cause of keratoconus is not known, but genetic and environmental factors are thought to be involved. Risk factors for developing keratoconus include younger age, allergic diseases such as hay fever, asthma and atopic dermatitis, and a positive family history. About one in 10 people with keratoconus also have a parent with the condition. Keratoconus can be triggered by eye rubbing, puberty or pregnancy.

Capter three: Symptoms?

Due to the protrusion of the cornea, an increasing short-sightedness, which is perceived as a deterioration of vision, develops. The protrusion of the cornea also causes an irregular curvature of the cornea, which causes further deterioration of vision due to image distortion. Affected people describe shadow vision and double contours. Often there is also a strong sensitivity to light. Since keratoconus has very different stages, the perceived symptoms and the time course of the disease vary greatly from person to person.

Capter four: Diagnosis?

Most of the time, the decrease in visual performance leads patients to an examination by an ophthalmologist. There, visual performance is measured and the surface of the eye is examined with the slit-lamp.

A special diagnostic test, called corneal topography, records images of your cornea and creates a detailed shape map of the corneal surface and posterior surface as well as the corneal thickness. Ultimately, this is how keratoconus is diagnosed these days. However, these examinations are not only essential for the diagnosis but also for the follow-up and thus the detection of changes.

Chater five: Treatment options?

Correction of the impaired vision is possible in the early stages of keratoconus with glasses, in more advanced stages only with rigid contact lenses. If contact lenses can no longer be fitted in very advanced stages, a corneal transplant can be performed to improve visual performance.

Stabilisation of the corneal tissue by collagen crosslinking (corneal crosslinking) can stabilise keratoconus in early stages in most cases or at least significantly slow down its progression. Results from follow-up periods of more than 10 years are available.

In general, it can be assumed that keratoconus does not lead to blindness. However, depending on the severity and the possibilities for correcting the visual performance, the disease can have a significant influence on your professional life. A cure for keratoconus is not yet possible.
