# Supplementary material for: The effect of a video tutorial to improve patients’ keratoconus knowledge – a randomized controlled trial and meta-analysis of published reports
Source: Front Ophthalmol (Lausanne). 2022 Oct 24;2:997257. doi: 10.3389/fopht.2022.997257 (PMC11182323; doi:10.3389/fopht.2022.997257)
Supplement: Additional file 2 — Study questionnaire. [file DataSheet_2.docx]

**Questionnaire**

In order to analyze and improve our information on the disease of keratoconus, we ask you to fill in this questionnaire immediately after the consultation with the attending physician and hand it in to our clark office. Thank you very much for your contribution and help!

**Knowledge on keratoconus**

Please answer each question with right or wrong:

1. **What is a keratoconus?**

☐ Right ☐ Wrong: Regular corneal astigmatism

☐ Right ☐ Wrong: Corneal protrusion

☐ Right ☐ Wrong: Eye disease, which deteriorates starting at the age of 35

☐ Right ☐ Wrong: Thickening of the cornea

☐ Right ☐ Wrong: Eye disease that only exists during puberty

1. **Are there risk factors for the occurrence of keratoconus?**

☐ Right ☐ Wrong: Other family members with keratoconus

☐ Right ☐ Wrong: Allergies protect against the occurrence

☐ Right ☐ Wrong: Contact lens wear

☐ Right ☐ Wrong: Occurs most frequently between the ages of 30 and 45

☐ Right ☐ Wrong: Atopy (such as neurodermitis)

1. **What are triggers for the onset of keratoconus?**

☐ Right ☐ Wrong: Eye rubbing

☐ Right ☐ Wrong: Contact lens wear

☐ Right ☐ Wrong: Pregnancy

☐ Right ☐ Wrong: Puberty

☐ Right ☐ Wrong: Frequent use of the smartphone

1. **What are symptoms of keratoconus?**

☐ Right ☐ Wrong: Light sensitivity

☐ Right ☐ Wrong: Perception of flashes

☐ Right ☐ Wrong: Vision deterioration

☐ Right ☐ Wrong: Foreign body sensation "like having dust in your eyes"

☐ Right ☐ Wrong: Letters are seen distorted

1. **What are consequences of untreated keratoconus?**

☐ Right ☐ Wrong: Vision correction with glasses is no longer possible

☐ Right ☐ Wrong: Visual performance without correction becomes worse

☐ Right ☐ Wrong: Contact lenses can no longer be fitted

☐ Right ☐ Wrong: Previous professional life can be strongly influenced

☐ Right ☐ Wrong: One goes blind

1. **What are treatment options for keratoconus?**

☐ Right ☐ Wrong: Spectacle adjustments bring no visual improvement

☐ Right ☐ Wrong: Soft contact lenses improve vision

☐ Right ☐ Wrong: Corneal transplant does not help

☐ Right ☐ Wrong: Corneal crosslinking improves vision

☐ Right ☐ Wrong: Hard contact lenses do not stop the progression of the disease

**Questions regarding your keratoconus**

1. **How many years ago were your diagnosed with keratoconus?**

Record years or year date: _________________

⬜ No specification

1. **What is your last completed level of education or which school are you currently attending?**

⬜ Compulsory school (upper secondary) ⬜ Foundation degree

⬜ GCE AS level (at age 17) ⬜ University

⬜ GCE A level (at age 18) ⬜ Professional degree program

⬜ Further education college ⬜ Not specified

1. **Have you ever had training in a medical field or have worked in a medical field?**

⬜ No ⬜ Yes -> please specify? ________________________________________________
